# Dietary assessment in intermittent fasting: validation of a short food frequency questionnaire vs. food records in diurnal dry fasting and time-restricted eating

**DOI:** 10.3389/fnut.2025.1552990

**Published:** 2025-07-28

**Authors:** Isabelle C. Schüssler, Christina L. Pappe, Christel von Scheidt, Beeke Peters, Henrik Dommisch, Christian Kessler, Andreas Michalsen, Daniela A. Koppold, Olga Pivovarova-Ramich

**Affiliations:** ^1^Department of Molecular Metabolism and Precision Nutrition, German Institute of Human Nutrition Potsdam-Rehbruecke, Nuthetal, Germany; ^2^TUM School of Life Sciences, Technische Universität München, Munich, Germany; ^3^Department of Periodontology, Oral Medicine and Oral Surgery, Charité – Universitätsmedizin Berlin, Corporate Member of Freie Universität Berlin and Humboldt-Universität zu Berlin, Berlin, Germany; ^4^Department of Internal Medicine and Nature-Based Therapies, Immanuel Hospital Berlin, Berlin, Germany; ^5^Institute of Social Medicine, Epidemiology and Health Economics, Charité – Universitätsmedizin Berlin, Corporate Member of Freie Universität Berlin and Humboldt-Universität zu Berlin, Berlin, Germany; ^6^Department for Prevention and Care of Diabetes, Department of Medicine III, Faculty of Medicine Carl Gustav Carus, Technische Universitä Dresden, Dresden, Germany; ^7^German Center for Diabetes Research (DZD), München-Neuherberg, Germany; ^8^Department of Endocrinology and Metabolism, Charité – Universitätsmedizin Berlin, Corporate Member of Freie Universität Berlin and Humboldt-Universität zu Berlin, Berlin, Germany

**Keywords:** intermittent fasting, religious fasting, time-restricted eating, validation, food frequency questionnaire, dietary assessment

## Abstract

**Objectives:**

Food frequency questionnaire (FFQ) is a cost-effective method of dietary assessment in nutritional and clinical research. It can be easily adapted to different research questions or populations, but modified versions require careful validation. This study assessed the validity of a short 14-item semi-quantitative FFQ compared to weighted food records in a secondary analysis of an intermittent fasting trial.

**Methods:**

Dietary assessment was conducted during the ParoFastin study, a controlled trial investigating the effects of religious Bahá’í fasting (19 days of diurnal dry fasting) and 16:8 time-restricted eating (TRE) on oral health and metabolic state compared to the habitual food intake. Daily consumption of meals, snacks, food groups, and overnight fasting time were assessed using both the short FFQ and food records. Food records were collected for 1 week at baseline and 19–21 days during the intervention and analyzed using PRODI^®^, a professional dietary assessment software. The FFQ was completed once at baseline and twice during the intervention. Its validity was assessed using correlation and method agreement analysis, including Bland–Altman plots for continuous data. Energy and macronutrient intakes were quantified using food records only.

**Results:**

Eight men and seven women, with a median age of 29 (27–34) years, were included in the validation analysis. Correlation coefficients ranged from 0.189 (tendency to snack) to 0.893 (meat consumption). Tendency to snack, frequency of snack consumption, and frequency of whole grain consumption showed insufficient agreement between the two methods. However, most questions of the short FFQ were found to be statistically valid in this population. According to food records, the energy, fat and carbohydrate intake were reduced during the Bahá’í fast and remained unchanged in the control and TRE groups compared to the baseline, while analysis of these parameters was not feasible based on the short FFQ.

**Conclusion:**

Overall, good agreement for the methods was found, although data on snack tendency, frequency of snack consumption, and whole-grain consumption were unreliable, indicating a need for questionnaire modifications. In contrast to time-consuming food records, the short FFQ can be effectively used in clinical trials and medical practice for specific goals.

**Clinical trial registration:**

https://drks.de/search/de/trial/DRKS00026701 German Clinical Trials Register (DRKS); identifier DRKS00026701.

## Introduction

The assessment of dietary behavior is an important aspect of nutritional and clinical research, with various methodologies already in use. Validation of a food frequency questionnaire (FFQ) as an assessment tool requires comparison with a reliable method, such as the gold standard for dietary assessment – the (weighing) food record. This research aims to statistically validate a new short FFQ by testing its agreement with food records. For this, we used nutritional data collected in a three-arm cohort study investigating the effects of 16:8 time-restricted eating (TRE) and intermittent religious dry fasting on oral health and metabolism (1, 2).

There are several methods for assessment and monitoring of dietary intake. Dietary survey methods may differ in the mode of administration (self- or interviewer-administered), the observed time frame (past, habitual, or current intake), the measurement of food quantities (weighing or estimating) and, if applicable, the conversion of foods to nutrients (3). Methods can also be distinguished according to whether they are prospective, recording diet directly at the time of consumption, or retrospective, asking respondents to recall their recent or usual dietary intake (3). Each dietary assessment method has its own set of advantages and limitations, making it the method of choice for different study settings.

Food frequency questionnaires query the frequency and amount of consumption of listed food items or food groups over a specified period of time, such as the previous week. They may be self-administered (paper- or web-based) or administered by an interviewer. Questions are either multiple choice or are open-ended regarding the intake frequency. The food items are generally grouped into categories that are similar in nutrient composition. The number and type of foods vary depending on the scope of research and may be culture or nutrient specific (3–5). FFQs can be used either to determine absolute intakes of foods or nutrients or to rank individuals by comparing their intakes to the mean intake of the study population. The latter is very useful in etiologic, epidemiologic studies investigating associations between dietary patterns and disease (6–9).

Food frequency questionnaires are cost-effective and characterized by a low burden on both investigators and respondents. They are easily adapted to different research aims and do not alter dietary behavior (3, 5, 10). Since they estimate the usual consumption over a longer period of time, it is not necessary to complete multiple FFQs. Despite some limitations such as reliance on memory, possible errors in portion size estimation, lower precision compared to other dietary assessment methods, and limited food item lists (3, 5, 10), FFQs is a valuable tool in dietary research, especially in large study populations. The content of FFQs should be tailored to their purpose and their target population. FFQs may be designed to assess intake of foods or food groups, or intake of nutrients. They may also include questions about general dietary habits, such as the number of meals (8).

It is crucial to validate a newly developed or adapted FFQ because even small changes can affect its validity. Otherwise, the resulting data may be incorrect and show misleading associations or, conversely, conceal existing associations between diet and disease (8, 11). In a study on different intermittent fasting regimens, we assessed the validity of a 14-item, semi-quantitative FFQ by comparing it to weighted food records collected throughout the study duration. The 14-item FFQ is a questionnaire developed at the Outpatient Clinic for Naturopathy of the Charité – University Medicine Berlin, which includes questions on general dietary habits, as well as the frequency of consumption of several food groups. It was developed as a short questionnaire for the use of clinicians to assess larger dietary changes in the clinical setting, and has also been applied in several small clinical studies such as ParoFastin trial described below.

## Materials and methods

### Study design

This study is a secondary analysis of the ParoFastin trial which was a three-arm, controlled study that explored the influence of two types of intermittent fasting, Bahá’í fasting (BF) and 16:8 TRE, on oral health and metabolism. Data on the bleeding on probing index, which was the primary outcome, and the 24-h glucose pattern were published previously (1, 2). The Bahá’í fast is a 19-day diurnal dry fast (12) in which participants do not consume any food or drink from sunrise to sunset. The fast is observed annually during the period leading up to the first day of the Bahá’í New Year, during the first weeks of March (13). This study was conducted at the Department of Periodontology, Oral Medicine and Oral Surgery, Charité – University Medicine Berlin, Germany, and the University Clinic for Dental Prosthetics of the Faculty of Medicine, Martin-Luther University Halle-Wittenberg, Germany, from November 2021 to May 2022. It was approved by the ethics committees of the Charité – University Medicine Berlin (ID: EA2/091/21) and Martin-Luther-University Halle-Wittenberg (2021-135) and registered at the German Clinical Trials Register (DRKS) under the identifier DRKS00026701. Eligible participants were between the age of 18 and 69 and were periodontally healthy with at least 24 teeth. For the BF group, it was required to be an official member of the Bahá’í community and intend to participate in the annual religious fast. Exclusion criteria have been listed elsewhere (2).

**FIGURE 1:**
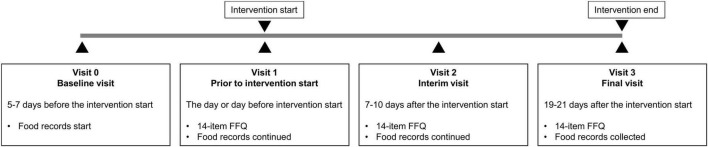
Study design. After baseline, participants either observed the Bahá’í fast or followed a 16:8 TRE regimen for 19 days or did not change their dietary habits (control group). The 14-item FFQ data were collected at three visits: prior to intervention start (V1) and twice during the intervention (V2 and V3). Throughout the entire study period, participants completed paper-based weighted food records.

After baseline, participants either observed the Bahá’í fast or followed a 16:8 TRE regimen for 19–21 days or did not change their dietary habits (control group) (Figure 1). The timing of the 8-h eating window could be chosen by the participants of the TRE group individually. Neither intervention group received dietary recommendations to modify their food choices. The 14-item FFQ data were collected at three visits: prior to intervention start (V1), after approximately 7–10 days of fasting (V2), and after approximately 19–21 days of fasting (V3) as described in detail elsewhere (2). In addition to the collection of metabolic and physiological data, all 66 participants completed one questionnaire on oral health-related quality of life, seven questionnaires on mental wellbeing, as well as the FFQ that was validated against food records in this analysis. The present analysis was conducted in a subcohort of 16 participants who agreed to complete paper-based weighted food records (together with simultaneous 24-h glucose monitoring) throughout the entire study period. Because the eating behavior was a secondary outcome of the ParoFastin study, no calculation of the sample size was conducted for this parameter.

### The 14-item food frequency questionnaire

The 14-item, semi-quantitative FFQ (Supplementary material 1) was developed approximately 15 years ago by Christel von Scheidt in the Outpatient Clinic for Naturopathy and Mind-Body Medicine (Immanuel Hospital Berlin and Charité – University Medicine Berlin) to assess the general dietary behavior of the clinic’s patients. The food list was developed on the basis of the Mediterranean whole-food diet with a reference period of the previous week. The FFQ contains questions on dietary habits, including the type of diet, whether snacks are consumed regularly, the number of meals and snacks consumed per day and the overnight fasting time. In the following section of the FFQ, the frequency of consumption of several food groups (vegetables, fruits, sausage, meat, cheese, confectionery, fast food, sweet beverages, and whole-grain products) is inquired. Portion sizes are provided.

### Food records

The participants were asked to document all consumed foods and drinks and the eating times during the baseline and fasting phases starting 5–7 days before fasting, comprising 24–28 days for each participant. They were instructed to weigh their food whenever possible, write down brand names, and use standard household measures (e.g., cups, glasses, tablespoon, teaspoon, etc.) when they go out for dinner. The food records were transcribed into PRODI^®^ 7.1, a professional dietary assessment software. In the case of imprecise information, such as the listing of dishes without specifying the exact ingredients or quantities, simple recipes were developed, or already existing recipes were taken from PRODI^®^. The weight of common food items, such as fruit, bread rolls, or sausages, was estimated using the *Monica-Mengenliste* (14). The nutrient composition of products from certain brands that are widely consumed in Germany was added to NutriBase^®^ if the product appeared in the food records. The next step was to extract all relevant information from the food records and the corresponding PRODI^®^ files to allow comparison with the FFQ data, e.g., foods were assigned to the food groups included in the FFQ. Nutritional data of one participant was completely excluded from the analysis because the dates of the food records did not match the periods the FFQ was answered for. For other 15 participants, all assessable days were included in the calculation of averages to increase accuracy. Reporting periods with less than 3 days of food records available were excluded from the analysis (*n* = 3), resulting in 42 analyzed data points (=15 participants in up to 3 recording periods each) for most items of the FFQ. Several parameters included less data points, i.e., number of snacks (*n* = 27, because this question could only be answered if participants stated that they regularly consumed snacks) and overnight fasting time (*n* = 40, because two questionnaires were assumed to contain an incorrect information).

### Statistical analysis

Statistical analysis was conducted using SPSS software v. 25. The normality of the data distribution was statistically tested using the Shapiro–Wilk test. Data are expressed as mean ± SD or median (interquartile range; IQR), according to the underlying data structure.

The responses regarding participants’ type of diet were statistically compared between the two methods using a McNemar–Bowker test of Symmetry. For the consumption of snacks, a McNemar’s test was performed. Additionally, the change over time for this question was tested using Cochran’s *Q* test. For continuous data, Wilcoxon signed-rank tests were conducted to test for differences between the two methods, because skewed parameters were revealed for some data sets. The effect size and interpreted using Cohen’s criteria (15). The α-level was set at 0.05 for all group difference tests.

As a mean of comparison of the two methods, data were correlated for each question of the FFQ. Cramér’s *V* was calculated for categorical, nominal data. The Phi coefficient was calculated for snack consumption. Spearman rank order correlation was used for continuous variables. In addition to the correlation coefficients *r*, the corresponding *p* values and coefficients of determination (*r*^2^) were determined. Correlation coefficients were interpreted according to Cohen (15).

The agreement between the two methods was analyzed using Bland–Altman plots for continuous data. A constant bias was considered systematic if its 95% confidence interval did not include the line of equality (*y = 0*) (16). Simple linear regression was performed to test for proportional bias. If a significant proportional bias and/or heteroscedasticity of data was found, data were log-transformed. In case of presence of zero values, a small constant (approximately half the smallest non-zero value) was added to all values before log-transformation. If a significant bias was still present in the adapted Bland-Altman plots, regression-based bias and limits of agreement were calculated according to the method of Bland and Altman (17). The Kappa Measure of Agreement was used instead of Bland-Altman analysis for categorical data.

The energy and macronutrients intake during baseline (V1) and intervention (combined V2 and V3 data) was assessed based on food records using PRODI^®^ software v. 7.1. In addition, the percentage of the total energy intake (EN%) was calculated for each macronutrient. Daily intake of energy in kilocalories and macronutrients in grams and EN% were compared between baseline and intervention for each of the three groups using paired-samples *t*-tests. In the case of non-normal distribution, data were transformed. In order to analyze the change across the three groups, one-way between-groups ANOVA were performed. The α-level was set at *p* = 0.05 for all tests. The effect size statistic provided is eta squared, which was interpreted according to Cohen (15). Equality of variances was tested using Levene’s test for Homogeneity of Variances. Gabriel’s post-hoc test was additionally performed in case of a significant ANOVA result (18).

## Results

### Study population

From totally 16 participants who completed both FFQ and food records, one participant was excluded from the analysis because as the dates of their food records did not align with the date of their FFQ completion. The remaining sub-cohort consisted of eight men and seven women with an average age of 29 (27–34) years and a BMI of 26.0 ± 4.1 kg/m^2^ at baseline. Six participants followed the Bahá’í fast, five were allocated to the TRE group, and four participants were allocated to the control group.

### Validation of the food frequency questionnaire

**FIGURE 2:**
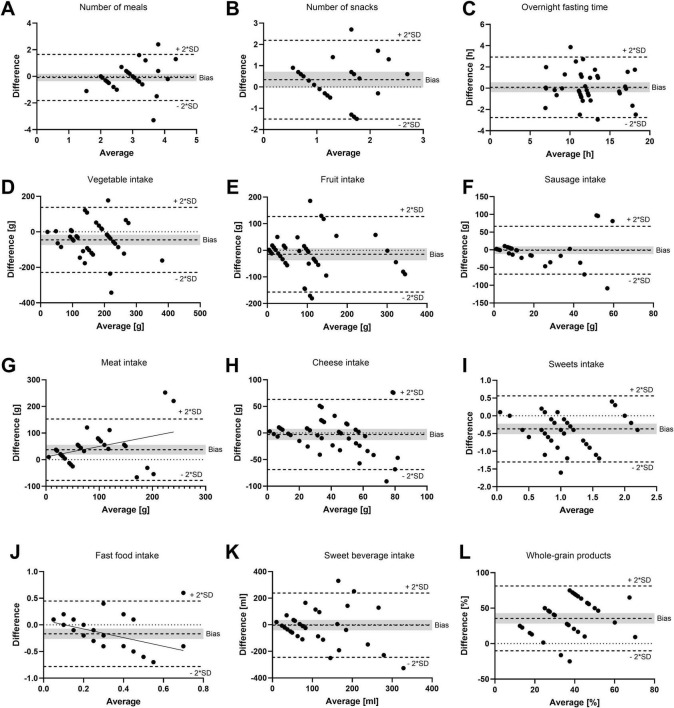
Bland–Altman plots as a measurement of method agreement. Difference between questionnaire and food record data plotted against the average value of the two methods. **(A)** Number of meals; **(B)** number of snacks; **(C)** overnight fasting time; **(D)** vegetable intake; **(E)** fruit intake; **(F)** sausage intake; **(G)** meat intake; **(H)** cheese intake; **(I)** confectionery (sweets) intake; **(J)** fast food intake; **(K)** sweet beverage intake; and **(L)** whole grain products. Notably, 95% limits of agreement (bias ±2*SD) as well as bias are shown as broken lines. The gray background indicates the 95% confidence interval of the bias. **(G,J)** Linear regression lines with significant slope (*p* < 0.05) are shown. **(A,D–L)** *N* = 42 data points = 15 participants in up to three recording periods. **(B)** *N* = 27 data points = 13 participants in up to three recording periods. **(C)** *N* = 40 data points = 15 participants in up to three recording periods.

**FIGURE 3:**
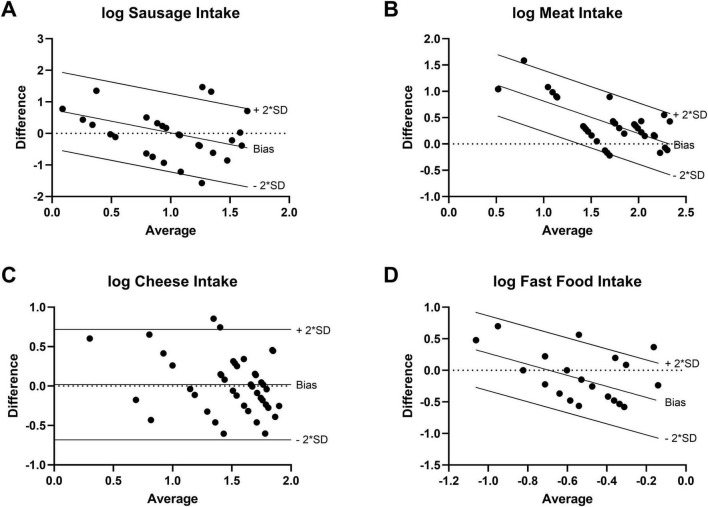
Transformed Bland-Altman plots to correct for severe heteroscedasticity or proportional bias (*N=42*). Difference between log-transformed questionnaire and food record data plotted against the logarithmic average value of the two methods. Regression-based Bland-Altman analysis in case of significant slope of linear regression line (*p* < 0.05). **(A)** Sausage intake [log(Y+0.5)]. **(B)** Meat intake [log(Y+1)]. **(C)** Cheese intake [log(Y+1)]. **(D)** Fast food intake [log(Y+0.05)].

For each study participant, data from three FFQs and three 24–28 food record days (one from baseline and two from the intervention period) were analyzed together. The validation study was performed using a combination of correlation and method agreement analyses. Adequate levels of agreement between the two methods were found for questions asking about the type of diet, the number of meals, the overnight fasting time as well as for the food categories of vegetables, fruits, sausage, meat, cheese, confectionery, fast food, and sweet beverages. Correlation coefficients for these questions ranged from 0.381 (sweet beverages) to 0.893 (meat), indicating moderate to strong correlations (Table 1). In Bland–Altman plots, the limits of agreement were within acceptable ranges for these questions (Figure 2). Conversely, the agreement between methods was found insufficient for the questions about the tendency to snack, the number of snacks, and the consumption of whole-grain products. For these questions, correlation coefficients were low [tendency to snack: 0.189 (*p* = 0.222); number of snacks: 0.205 (*p* = 0.304); and whole-grain products: 0.280 (*p* = 0.073)] and the 95% areas of agreement in Bland–Altman plots were considered too wide. Systematic underestimation was found for vegetables, confectionery, and fast food, while meat and whole-grain products were systematically overreported. Heteroscedasticity was especially severe for the food groups of sausage, meat and cheese, and a significant proportional bias was detected for fast food and meat. Log-transformation (log(Y+1)) before analysis and a following back-transformation resulted in a mean bias of 1.04 (i.e. overestimation by 4%) for cheese (Figure 3C). For a given intake level, 95% LoA ranged approximately from 20% to 520%, indicating wide variability of data. For sausage, meat and fast food, log-transformation resulted in homoscedasticity but regression-based Bland-Altman analyses were performed because proportional bias was detected (Figure 3). For fast food, the bias indicated increasing underreporting with the average intake (Figure 2J). In contrast, absolute levels of overreporting increased with the average meat intake (Figure 2G), while overreporting actually decreased relative to mean meat intake (Figure 3B).

**TABLE 1:** Daily consumption of meals, snacks, and food groups and overnight fasting time according to the food frequency questionnaire (FFQ) and food records (FR).

Validation items	FFQ	FR	Correlation	*p*-Value*
	Median (IQR)	Median (IQR)	Rho (ρ)	
Number of meals	3.0 (2.0–3.0)	2.8 (2.5–3.2)	0.486 (0.001)	0.345
Number of snacks^a^	1.0 (1.0–2.0)	1.1 (0.6–1.6)	0.205 (0.304)	0.045
Overnight fasting time^b^	12.0 (11.0–15.5) h	12.1 (9.7–16.0) h	0.851 (<0.001)	0.968
Vegetables	150.0 (78.6–200.0) g	193.8 (122.2–240.9) g	0.439 (0.004)	0.002
Fruits	64.3 (21.4–100.0) g	81.6 (29.4–157.5) g	0.604 (<0.001)	0.152
Sausage	2.5 (2.5–10.7) g	3.2 (0.0–20.5) g	0.483 (0.001)	0.637
Meat	37.5 (37.5–137.5) g	32.6 (1.7–65.0) g	0.893 (<0.001)	<0.001
Cheese	45.8 (12.5–58.3) g	39.6 (19.4–57.6) g	0.428 (0.005)	0.644
Confectionery	0.8 (0.5–1.0)	1.2 (0.9–1.7)	0.661 (<0.001)	<0.001
Fast food	0.2 (0.1–0.2)	0.35 (0.1–0.7)	0.451 (0.003)	0.002
Sweet beverages	70.7 (20.0–165.0) ml	66.0 (0.0–144.0) ml	0.381 (0.014)	0.773
Whole grains	50.0 (25.0–75.0)%	9.6 (0.0–24.1)%	0.280 (0.073)	<0.001

Methods were compared in Wilcoxon signed-rank tests. Data are presented as median (IQR). Spearman rank order correlation coefficients rho are given with corresponding *p* values. For all parameters, *N* = 42;

^a^*N* = 27;

^b^*N* = 40 data points.

*p-Value corresponds to test for differences between methods (Wilcoxon signed-rank test).

### Comparison of results of FFQ and food records

**FIGURE 4:**
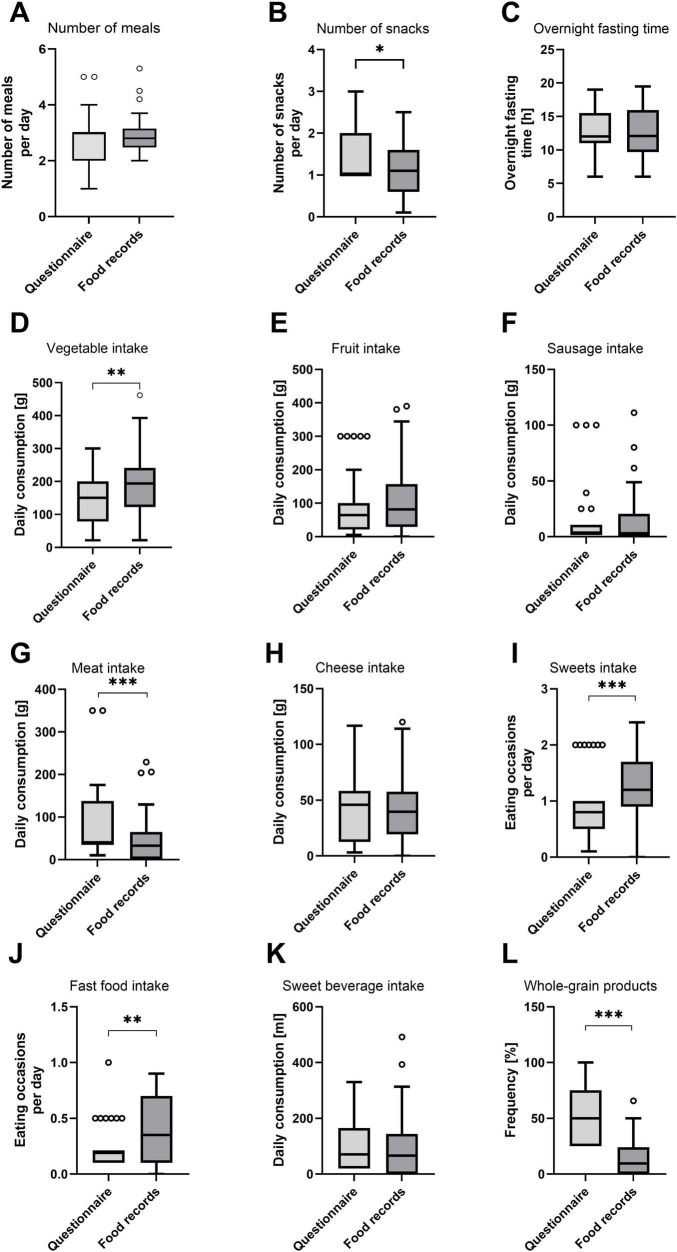
Average daily number of meals and snacks, overnight fasting time, and consumption of food groups according to FFQ and food records. **(A)** Number of meals; **(B)** number of snacks; **(C)** overnight fasting time; **(D)** vegetable intake; **(E)** fruit intake; **(F)** sausage intake; **(G)** meat intake; **(H)** cheese intake; **(I)** confectionery (sweets) intake; **(J)** fast food intake; **(K)** sweet beverage intake; and **(L)** whole grain products. Visualization of FFQ and food record data as Tukey boxplots. Circles represent outliers. Differences were statistically tested using a Wilcoxon signed-rank test. Stars indicate significance (*p < 0.05; **p < 0.01; ***p < 0.001). **(A,D–L)** *N* = 42 data points = 15 participants in up to three recording periods. **(B)** *N* = 27 data points = 13 participants in up to three recording periods. **(C)** *N* = 40 data points = 15 participants in up to three recording periods.

On average, 3.0 (2.0–3.0) and 2.8 (2.5–3.2) meals were consumed according to FFQ and food record data, respectively (Figure 4A). According to the FFQ, participants consumed 1.0 (1.0–2.0) snack per day, while they consumed 1.1 (0.6–1.6) snacks according to food records. The median value for the estimated time that passed between the last meal of 1 day and the first meal of the next day (overnight fasting time) was 12.0 h (11.0–15.5 h) in the FFQ and 12.1 h (9.7–16.0 h) according to the food records. Consumption frequencies of the food groups included in the FFQ are also visualized in Figure 4. The results of the Wilcoxon signed-rank tests testing for statistical differences between the methods confirmed the systematic differences that were found in the Bland–Altman plots. Additionally, statistically different results between methods were determined for the number of snacks, as shown in Figure 4B. The question about the consumption of whole-grain products resulted in particularly large inter-method differences: Participants reported consuming whole-grain products half of the time (25.0%–75.0%) in the FFQ, while they documented a frequency of only 9.6% (0.0%–24.1%) in the food records.

### Daily energy and macronutrient intake

**FIGURE 5:**
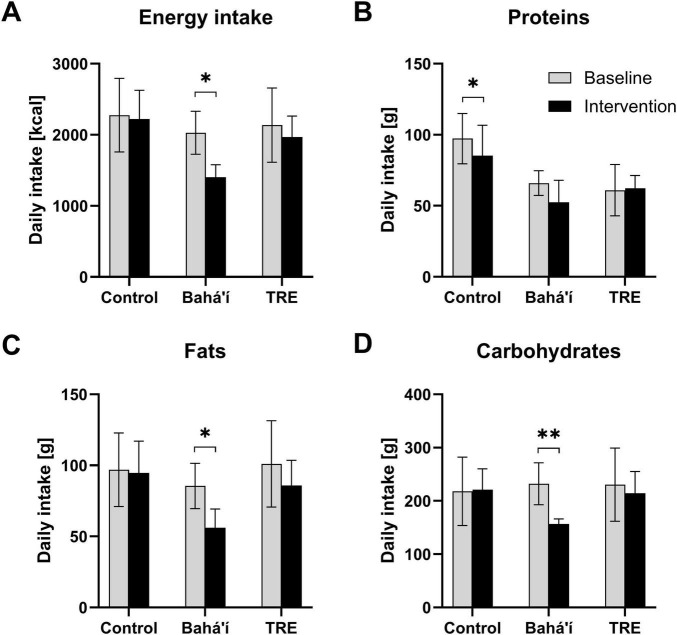
Daily consumption of energy **(A)** and the macronutrients proteins **(B)**, fats **(C)**, and carbohydrates **(D)** derived from food records. Bar diagrams illustrating mean daily intake for the three different groups, control (*N* = 4), Bahá’í (*N* = 5), and time-restricted eating (TRE; *N* = 3), during baseline and intervention. Error bars represent standard deviations. Three participants were excluded from this analysis because baseline data were not available. Comparison of the nutrient intake during baseline (V1) and intervention (V2 and V3) per group using paired-samples *t*-tests (*p < 0.05; **p < 0.01).

The energy and macronutrient intake were analyzed based on the food records while this was not feasible based on the FFQ. There was no significant change in energy intake in the control as well as the TRE group (Figure 5). However, there was a decrease by 625.5 ± 425.7 kcal in the Bahá’í group between baseline and intervention (*p* = 0.030). The daily fat and carbohydrate intake declined by 29.4 ± 23.4 g (*p* = 0.048) and 75.2 ± 42.3 g (*p* = 0.007), respectively, within the Bahá’í group only. Daily protein intake declined by 12.0 ± 4.4 g within the control group (*p* = 0.013). The distribution of proteins, fats and carbohydrates as percentage of the total energy intake through macronutrients (EN%) did not change between baseline and intervention for any group (Table 2).

**TABLE 2:** Daily intake of energy and macronutrients in EN% in the three study groups based on food record data.

Variable	*N*	Statistical parameters	Proteins	Fats	Carbohydrates
Control	4	B [%]	18.83 ± 4.12	40.35 ± 4.58	40.81 ± 6.45
		I [%]	16.45 ± 2.20	40.44 ± 4.23	43.12 ± 5.97
		Δ [%]	−2.39 ± 2.45	0.08 ± 2.51	2.30 ± 2.31
		*p*-Value	0.135	0.951	0.140
		Eta squared	0.579	0.001	0.570
Bahá’í	5	B [%]	13.73 ± 2.64	38.71 ± 2.54	47.56 ± 3.71
		I [%]	15.64 ± 3.93	36.92 ± 4.38	47.44 ± 4.84
		Δ [%]	1.90 ± 2.27	−1.79 ± 4.63	−0.12 ± 4.72
		*p*-value	0.114	0.437	0.959
		Eta squared	0.504	0.157	0.001
TRE	3	B [%]	11.74 ± 0.57	43.29 ± 7.61	44.97 ± 7.66
		I [%]	13.38 ± 1.14	40.64 ± 5.50	45.98 ± 6.56
		Δ [%]	1.64 ± 1.20	−2.65 ± 3.80	1.02 ± 2.70
		*p*-value	0.130	0.350	0.582
		Eta squared	0.757	0.422	0.174

EN% is the percentage of the total energy intake via macronutrients. Δ represents the difference between intervention and baseline. Data are given as mean ± SD. Changes between baseline (B) and intervention (I) were statistically analyzed using paired sample *t*-tests for each study group (alpha level = 0.05). The effect size provided is eta squared.

## Discussion

The short 14-item FFQ demonstrated predominantly good agreement with the food records in this validation analysis and, with the exception of the categories “snacks” and “whole-grain products,” could be used to evaluate short-term dietary changes in specific cohorts. However, the severe heteroscedasticity in some food categories indicates that respondents who consumed these foods frequently had greater difficulty accurately estimating their intake compared to those who consumed them rarely or not at all.

The question whether participants tend to snack between meals is very subjective since there is no frame or limit to the frequency of consumption given. The personal definition of a tendency to snack could differ between participants and be the reason for the low validity of this question. It would be beneficial to remove this question and instead ask all participants to indicate the number of snacks they consume on a regular basis. This may also increase the validity of the question about the number of snacks participants consumed daily, which so far has only been accessible to participants who had reported to tend to snack in the question before. Participants highly overestimated their intake of whole-grain products and the results of this FFQ question are considered imprecise and invalid. A likely reason for this is that respondents did not have a thorough understanding of the definition of whole-grain products. Multigrain products, which contain more than one type of grain, are common in Germany and may be confused with whole-grain products by respondents. However, multigrain products do not necessarily contain whole grains and thus may not have the same nutritional qualities as whole-grain products (19, 20). In contrast, food records provide more information on the type of food, especially due to the possibility to documenting branded products, whereby the exact nutrient composition can be determined. Indeed, the short FFQ requires an explanation of what whole grain products are and needs underlined with concrete examples in order to achieve a better understanding. Whole-grain products, should be preferred over refined grains (21). These have been associated with reduced risk of cardiovascular disease, type 2 diabetes mellitus, and cancer, and may benefit gastrointestinal health (22–24). The high discrepancy between food records and FFQ in this validation study suggests that respondents are aware of the beneficial health effects of these products and want to consume them but may lack knowledge about which foods are covered by the term whole-grain. Campaigns to increase knowledge about whole-grain foods could therefore have a positive effect on the frequency of consumption of this food category. The respondents in this validation study reported consuming a total amount of 214.3 g of fruit and vegetables in the FFQ, and 275.4 g according to the food records. This is slightly lower than the results of the German National Nutrition Survey (NVS II), the most recent representative consumption survey of the German population, and its follow-up study NEMONIT (25–27) and amounts to about 40%–50% of the amount recommended by the German Nutrition Society (DGE) depending on the dietary assessment method (28). However, shortened eating windows in intermittent fasting regimens may play an important role regarding this discrepancy and may lead to a lower vegetable and fruit consumption. Further, intermittent fasting was performed in early spring when availability seasonal of fresh fruits and vegetables was limited. This may support the feasibility of both dietary assessment methods in the here studied cohort regarding the vegetable and fruit category.

Data for meat category in FFQ revealed differences vs. food records and therefore the question regarding this category should be revised in order to achieve understandability for the person who is completing the form. Overall, data from short FFQ and food records reveal that participants in this validation study met current DGE guidelines for meat and meat product intake (28). In NVS II and NEMONIT, the consumption values are considerably higher (25, 27). One possible explanation for the low consumption of meat and meat products by this sub-cohort is that people who volunteer to participate in a fasting study are likely to be focused on their health and may consume reduced amounts of meat as a result. In addition, some members of the Bahá’í group reported that they completely abstained from all food products of animal origin for the duration of the fast. The Bahá’í teachings do not forbid meat consumption, but it is believed that abstinence from meat enhances spiritual qualities (29). The general eating habits of the Bahá’í community, however, require further investigation. According to a representative survey conducted in 2023, 9% of Germans are vegetarians, while 3% report following a vegan diet (30). Conversely, one third of the participants in this validation study reported to have eaten no meat during the reporting period of the study, based on both FFQ and food record data. Therefore, the percentage of vegetarians and vegans in this subset trial was substantially higher than in the general population in Germany. These trends might have contributed to increased variability in measurement error and hence heteroscedasticity. Daily cheese consumption in this sub-cohort was comparable to the results of the NVS II diet history interviews (26). The DGE currently recommends consuming two portions of dairy products per day. One portion can be 250 g of milk, 150 g of yogurt, or 30 g of cheese (28). Thus, more than one portion of dairy is already covered by cheese in this study, indicating that the total amount of dairy products consumed may be higher than recommended by the DGE.

Data regarding confectionary and fast food consumption show high accuracy between the validated short FFQ vs. food records. Fast food consumption, however, was significantly underreported for high intakes, which is indicated by a significant proportional bias. In particular these categories are highly relevant with regard to diabetes prevention in the modern society. However, although a comparison of confectionery and fast food consumption in this study to recent data was not possible, both food categories should be limited due to adverse health effects, as recommended by the DGE (21). The consumption of fruit juices and soft drinks was considerably lower in this sub-cohort than in NVS II (25). The DGE currently recommends the consumption of two glasses of 200 g of fruit and vegetable juices per week (28). The intake of sugar-sweetened beverages should be avoided altogether to limit adverse health effects according to the DGE (21).

The energy and macronutrient intake are crucial parameters for accurately assessing dietary adherence in nutritional intervention trials, including intermittent fasting studies (31). Notably, the assessment of energy and macronutrient intake in this study was not feasible using the short FFQ. Analysis, based on food records and conducted with PRODI software, revealed no significant change in energy intake within the control and TRE groups consistent with previous FDDB-based assessment (2). Remarkably, the energy intake as well as the absolute (in gram) fat and carbohydrate intake were reduced during the intervention compared to baseline within the Bahá’í group only. Our study provides the first research data on food intake during the Bahá’í fast, whereas data in larger cohorts is lacking. For Ramadan fasting, another form of religiously motivated daytime dry fasting, conflicting evidence exists, with some studies reporting increased energy and fat intakes while others report decreases. In a systematic review, Osman et al. (32) conclude that dietary habits and meal composition differ between cultures and countries, which may explain the conflicting results. Further research on the dietary behavior of intermittent dry fasters should be considered.

All in all, this FFQ validation study has several important strengths. It uses both correlation analysis and method agreement, mostly Bland–Altman plots, to analyze the validity of the FFQ, thus combining two very different validation methods. As the reference method, food records are used. The combination of a prospective and a retrospective dietary assessment method, food records and FFQ respectively, results in a reduction of shared measurement error, thus increasing the accuracy of the validation analysis.

There are also several limitations to this validation study. First and foremost, the sample size of this pilot study is relatively small due to limited resources. Nevertheless, the analyzed dataset was larger including three FFQs and 24–28 food record days for each individual. Although a possible learning bias due to repeated FFQ measure have to be mentioned, the statement on this is controversial. Indeed, Cade et al. (8) even emphasized the potential of two measurements in one subject for improvement of precision which therefore can be considered as a strength, and not as a limitation. Second, the study participants were self-selected volunteers who may already have a heightened awareness of their dietary habits. This may result in more accurate responses and, thus, bias the validity analysis (12). Another limitation is the concurrent keeping of food records which may introduce a bias by leading to an increased focus on diet (6). The FFQ has a comparatively short food list, missing several food groups that may contribute significantly to energy and nutrient intakes, such as dairy products, eggs, or legumes. In addition, the food groups are too broad and unspecific, for example, there is no distinction between red meat and poultry. Some food group definitions also overlap. An example is ultra-processed meats such as bratwurst, which should be included in both the sausage and fast food categories. Therefore, this FFQ is not suitable for nutrient analysis.

## Conclusion

In conclusion, the statistical validation of this short FFQ is an essential step in its development and is required before it can be used regularly in clinical settings and research. Except for the three questions with poor agreement between methods, this FFQ can be considered an appropriate tool for assessing the intake of specific food groups after an additional validation in a larger cohort.

## Data Availability

The raw data supporting the conclusions of this article will be made available by the authors, without undue reservation.
