# Supplementary material for: Dietary assessment in intermittent fasting: validation of a short food frequency questionnaire vs. food records in diurnal dry fasting and time-restricted eating
Source: Front Nutr. 2025 Jul 28;12:1552990. doi: 10.3389/fnut.2025.1552990 (PMC12336244; doi:10.3389/fnut.2025.1552990)
Supplement: Supplementary file 1 [file Table_1.docx]

**Supplement 1: Short Food Frequency Questionnaire** (translated from German to English)

What best describes your form of diet?

Including meat and fish

Including meat but not fish

Including fish but not meat

Vegetarian with dairy products and eggs

Vegan without any animal products

Vegetarian with dairy products, but not eggs

Other, please specify _______________________

How many meals (including small meals) did you consume per day on average in the last week?

One meal

Two meals

Three meals

Four meals

Five meals

More than five meals

Meals include the usual main meals breakfast, lunch, and dinner, irrespective of the time at which they are consumed.

Small meals include fruit, vegetable sticks, nuts, yoghurt, but also dishes such as sandwiches, if these are not components of a main meal. Chocolate bars, sweets, candy, potato chips etc. are not regarded as small meals.

Have you tended to snack between meals in the last week? (Chocolate bars, sweets, pastries, potato chips etc.)

Yes

No

How many portions of snacks do you eat throughout the day?

Answer this question only if the following condition is fulfilled:

Response was “Yes” for question “Have you tended to snack between meals in the last week? (Chocolate bars, sweets, pastries, potato chips etc.)”

Only numbers may be entered. Please enter your response here:

1 Portion = 1 Hand full or 1 chocolate bar

How many hours on average passed in the last week between the last and the first time you ate?

Only numbers may be entered. Please enter your response here:

Example: You eat your dinner at 7 p.m. but usually have a snack while watching television around 9 p.m. The next morning you have breakfast at 7 a.m. Therefore, 10 hours passed between the last and the first time you ate.

In the past week, about how many portions of vegetables did you eat?

3 portions per day or more

2 portions per day

1 portion per day

5-6 portions per week

3-4 portions per week

1-2 portions per week

Rarely or not at all

1 portion = about 100 g

Examples: 1 big tomato / ¼ of a cucumber / 1 bowl of salad

In the past week, about how many portions of fruit did you eat?

3 portions per day or more

2 portions per day

1 portion per day

5-6 portions per week

3-4 portions per week

1-2 portions per week

Rarely or not at all

1 portion = about 100 g

Examples: 1 small apple / 1 small banana

In the past week, about how many portions of sausage did you eat?

3 portions per day or more

2 portions per day

1 portion per day

5-6 portions per week

3-4 portions per week

1-2 portions per week

Rarely or not at all

1 portion = 2 slices of 20-30 g each

In the past week, about how many portions of meat did you eat?

3 portions per day or more

2 portions per day

1 portion per day

5-6 portions per week

3-4 portions per week

1-2 portions per week

Rarely or not at all

1 portion = 150-200 g meat

In the past week, about how many portions of cheese did you eat?

3 portions per day or more

2 portions per day

1 portion per day

5-6 portions per week

3-4 portions per week

1-2 portions per week

Rarely or not at all

1 portion = about 2 slices of 20-30 g each / half a mozzarella / ¼ of a bag of grated cheese

In the past week, how often did you consume confectionery? (Cake, ice cream, chocolate…)

3 times per day or more

2 times per day

Once per day

5-6 times per week

3-4 times per week

1-2 times per week

Rarely or not at all

In the past week, how often did you consume fast food? (French fries, burger, pizza, bratwurst)

3 times per day or more

2 times per day

Once per day

5-6 times per week

3-4 times per week

1-2 times per week

Rarely or not at all

In the past week, how many glasses of sweetened beverages, soft drinks or juices did you drink?

3 glasses per day or more

2 glasses per day

1 glass per day

5-6 glasses per week

3-4 glasses per week

1-2 glasses per week

Rarely or not at all

1 glass = about 330 ml Coca Cola, lemonade, Fanta, fizzy drink, juices, iced tea

In the past week, how often did you eat whole-grain products? (Bread, rice, pasta)

Exclusively

Mainly

About half the time

Occasionally

Rarely or never
